# Gene Expression Profile of Peripheral Blood Monocytes: A Step towards the Molecular Diagnosis of Celiac Disease?

**DOI:** 10.1371/journal.pone.0074747

**Published:** 2013-09-17

**Authors:** Martina Galatola, Valentina Izzo, Donatella Cielo, Marinita Morelli, Giuseppina Gambino, Delia Zanzi, Caterina Strisciuglio, Maria Pia Sperandeo, Luigi Greco, Renata Auricchio

**Affiliations:** 1 Department of Translational Medical Science, University of Naples Federico II, Naples, Italy; 2 European Laboratory for Food-Induced disease (ELFID), University of Naples Federico II, Naples, Italy; 3 Department of Molecular Medicine and Medical Biotechnology, University of Naples Federico II, Naples, Italy; Centro di Riferimento Oncologico, IRCCS National Cancer Institute, Italy

## Abstract

**Aim:**

Celiac disease (CD) is a multifactorial autoimmune disease induced by ingestion of gluten in genetically predisposed individuals. Despite technological progress, the diagnosis of CD is still based on duodenal biopsy as it was 50 years ago. In this study we analysed the expression of CD-associated genes in small bowel biopsies of patients and controls in order to explore the multivariate pathway of the expression profile of CD patients. Then, using multivariant discriminant analysis, we evaluated whether the expression profiles of these genes in peripheral blood monocytes (PBMs) differed between patients and controls.

**Participants:**

Thirty-seven patients with active and 11 with treated CD, 40 healthy controls and 9 disease controls (Crohn’s disease patients) were enrolled.

**Results:**

Several genes were differentially expressed in CD patients versus controls, but the analysis of each single gene did not provided a comprehensive picture. A multivariate discriminant analysis showed that the expression of 5 genes in intestinal mucosa accounted for 93% of the difference between CD patients and controls. We then applied the same approach to PBMs, on a training set of 20 samples. The discriminant equation obtained was validated on a testing cohort of 10 additional cases and controls, and we obtained a correct classification of all CD cases and of 91% of the control samples. We applied this equation to treated CD patients and to disease controls and obtained a discrimination of 100%.

**Conclusions:**

The combined expression of 4 genes allows one to discriminate between CD patients and controls, and between CD patients on a gluten-free diet and disease controls. Our results contribute to the understanding of the complex interactions among CD-associated genes, and they may represent a starting point for the development of a molecular diagnosis of celiac disease.

## Introduction

Celiac disease (CD) is gluten-induced autoimmune disease closely linked to a specific genetic profile. More than 95% of celiac patients are HLA-DQ2/8 carriers, although HLA genes account for only around 35% of the genetic variation [1,2]. A recent genome-wide association study identified 13 known, 13 new and 13 “suggested” genomic variants [3]. Although these 39 risk variants account for less than 15% of the genetic variance, they help to shed light on the immunological factors involved in the gluten-induced abnormal response (i.e., T-cell development, innate immune detection of viral RNA, T- and B-cell co-stimulation/inhibition and cytokines, chemokines and their receptors) [3,4]. But much more work is needed to explain the missing heritability and to understand the functional consequences of associated alleles in this complex disease.

Based on expression quantitative trait meta-analysis, Dubois et al. identified celiac risk variants correlated with *cis* gene expression in 20 out of 38 (52.6%) tested loci [3]. A study of gene expression in duodenal mucosa of a Spanish celiac sample [5] confirmed most of the results reported by Dubois et al.

In a previous study [6], we evaluated the expression of genes clustered on chromosome 4q27 (*KIAA1109*, *IL-2* and *IL-21*) and of the *c-REL* gene in intestinal mucosa of controls and CD patients (active and treated with a gluten-free diet [GFD]). *KIAA1109* and *c-REL* mRNA expression in intestinal mucosa was significantly higher in CD patients on GFD than in CD patients on gluten. Another study found that IL-21 expression was higher in CD patients than in controls [7]. This increase seems to be gluten-dependent because IL-21 expression returned to the levels of controls after at least one year of GFD [6]. In the same study, *IL-2* mRNA did not differ among CD patients, CD-GFD patients and controls, although there was a trend towards over-expression in the CD group.

We recently analysed these genes in a family cohort association study [8]. Similarly we explored their contribution to the progression of the potential CD phenotype [6]. These mono-dimensional observations of the expression of single genes do not provide a valid picture of the complex inter-relationship of these molecules at cellular level: a multivariate approach is needed because the expression of each gene cannot be independent from the expression of the other genes in a functional pathway.

A discriminant analysis of gene expression was recently proposed as a promising diagnostic tool to distinguish celiac atrophic mucosa from normal mucosa [9]. As expected, genes involved in the alteration of the crypt-villi architecture in the small intestinal mucosa were identified, and they matched the histological alterations.

The aim of this study was to explore the expression of genes associated to CD in the target tissue in order to estimate the contribution of each single gene to the development of the gluten-induced immune response. Then, using a multivariate model, we planned to evaluate the same set of genes in peripheral blood monocytes (PBM). The rationale for using PBMs is that they are more readily available than mucosal tissue: monocyte-derived cells (MDCs) were found to accumulate in the inflamed intestine of CD patients [10,11]. In a recent study, it was shown that the density of CD14+ CD11c+ MDCs was increased in the inactive form of the disease whereas the density of CD14- cells and macrophages was decreased in the active celiac lesion [12].

## Results

### Expression of the candidate genes in duodenal mucosa

Duodenal intestinal mucosa samples of controls, CD and CD-GFD patients were examined for the expression of *KIAA1109, IL-2, IL-21, LPP, RGS1, cREL, SH2B3, TAGAP, TNFAIP3, TNFSF14, and TNFRSF14* genes, according to our previous results [8]. The expression of the *LPP* gene did not differ among the three groups (Figure S1 A). *TNFAIP3 and RGS1* mRNA levels were up-regulated in CD patients versus controls but the difference was not statistically significant (Figure S1 B and C). Interestingly, *TNFRSF14* mRNA levels in duodenal mucosa were similar in controls and CD-GFD patients, and higher, albeit not significantly so, in CD patients (Figure S1 D).

**Figure 1:**
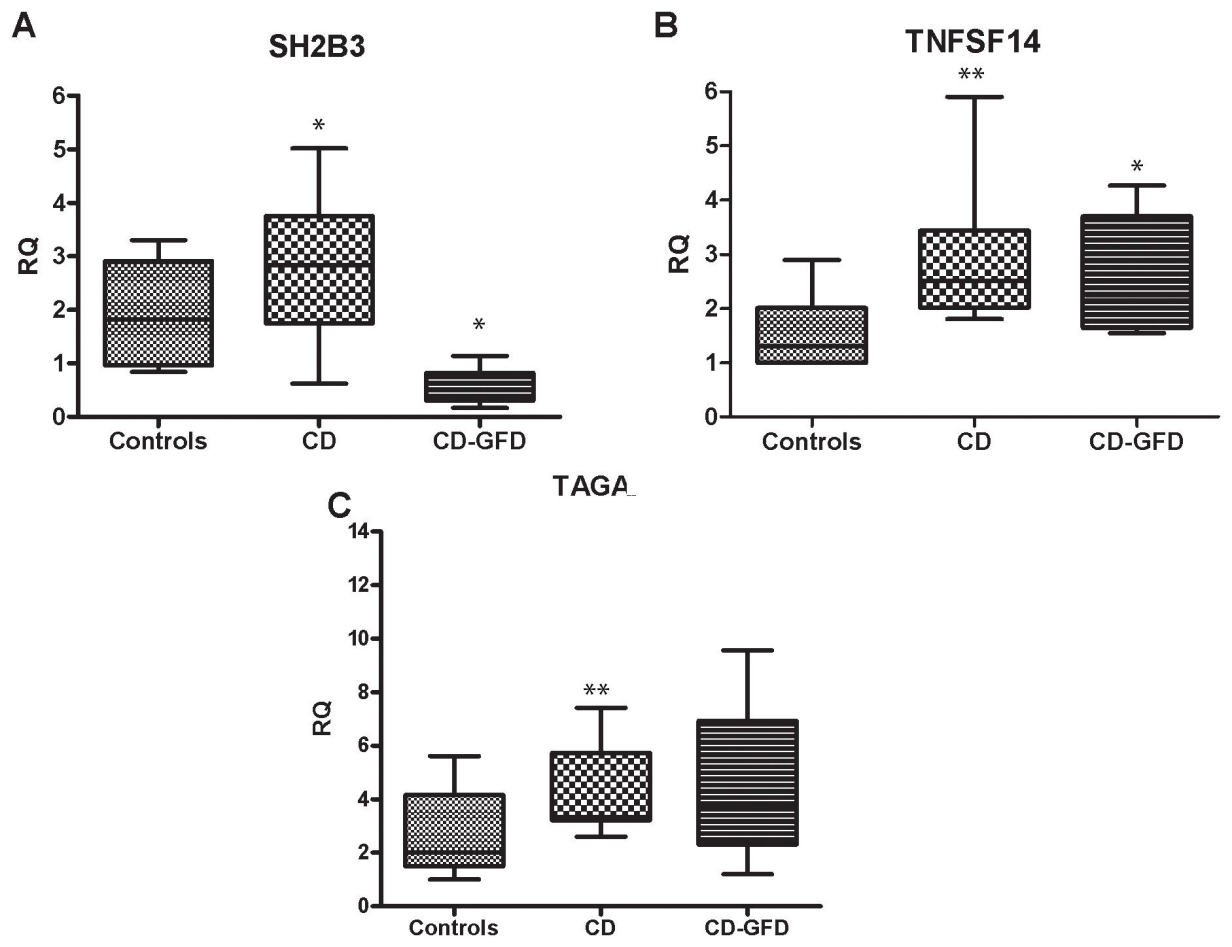
mRNA expression of new candidate genes in duodenal tissue. **A**) *SH2B3* expression was slightly higher in CD patients than in controls, and significantly lower in CD-GFD patients than in controls (*p*<0.01) and CD patients (*p*<0.01); **B**) *TNFSF14* expression was higher in CD versus controls, and remained higher also after one year of GFD versus controls (*p*=0.04) and CD patients (*p*<0.01); **C**) *TAGAP* expression was higher in CD patients versus controls (*p*=0.04);. RQ: relative quantification; Ctr: controls; CD: celiac disease; CD-GFD: celiac patients on a gluten-free diet; * *p*<0.01, **p<0.05.

**Figure 2:**
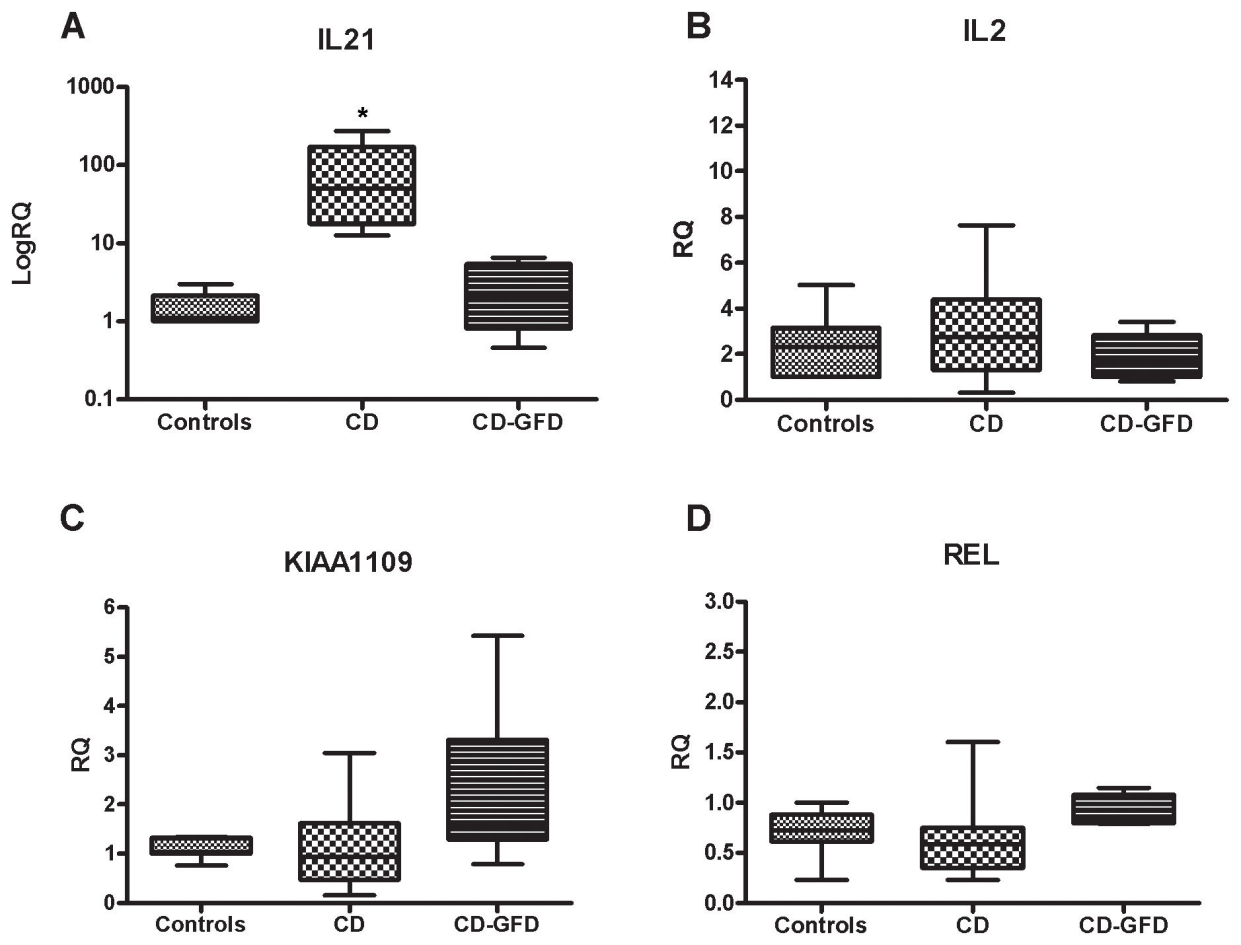
Analysis of the levels of mRNA expression of associated genes in duodenal tissue. A) IL-21 is over-expressed in CD compared to controls (*p*<0.01), B) IL-2 shows a very small trend of increase in CD compared to controls; C) and D) Expression of the KIAA1109 and cREL genes: the patterns are very similar and did not show any variations among the three groups. RQ: relative quantification; Ctr: controls; CD: celiac disease; CD-GFD: celiac patients on a gluten-free diet; * *p*<0.01, **p<0.05.

*SH2B3* expression was higher in CD mucosa than in control mucosa, and significantly lower in CD-GFD subjects than in either controls or CD patients (Figure 1 A). Mucosal *TNFSF14* levels were higher in CD patients and in CD-GFD patients than in controls (Figure 1 B). *TAGAP* expression was significantly higher in CD mucosa than in control mucosa (Figure 1 C). As we expected, the expression levels of *IL-21* were significantly higher in CD than in controls (*p*<0.01), which confirms our previous data (Figure 2 A) [8]. On the contrary, *IL-2, KIAA1109 and cREL* mRNA did not differ between the two groups, only IL-2 shows a very small trend (although not significant) of increase in CD compared to controls (Figure 2 B, C and D).

### Expression of the candidate genes in peripheral blood monocytes

The expression of the panel of candidate genes was evaluated in monocytes extracted from peripheral blood samples of 18 controls, 17 CD and 5 CD-GFD patients. Monocytes extracted from peripheral blood of 9 Crohn patients served as positive controls. We did not evaluate the expression of *IL-2 and IL-21* because they are not produced by monocytes but by CD4+ T-cell lines TCLs after antigen activation.

**Figure 3:**
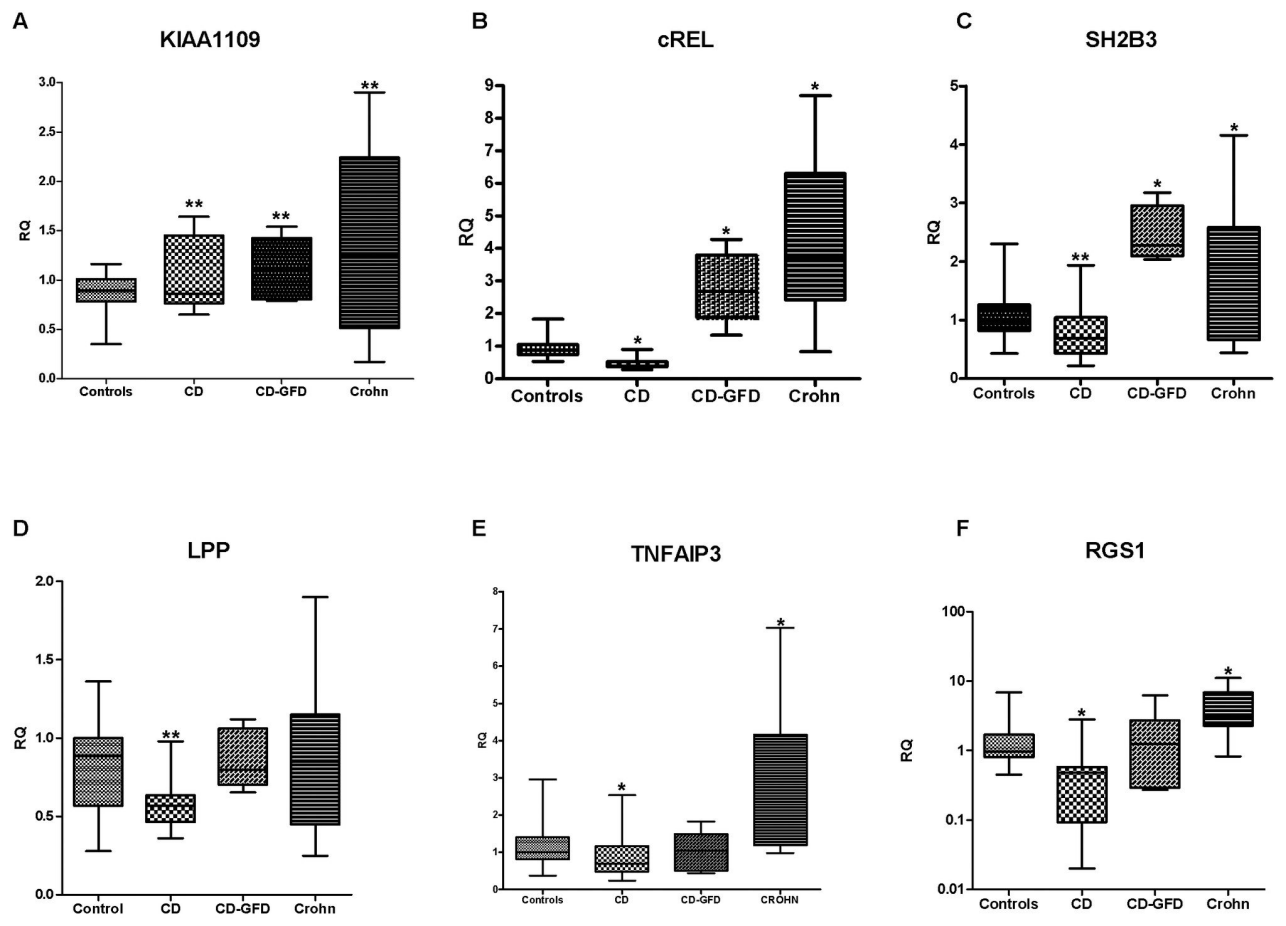
mRNA expression of candidate genes in peripheral blood monocytes. A) *KIAA1109*expression was higher in CD (*p*=0.05) and in CD-GFD patients (*p*=0.05) than in controls, but also in Crohn peripheral monocytes (*p*=0.02); B) *c-REL* expression was lower in CD peripheral monocytes than in controls (*p*<0.01), but become higher than controls (*p*<0.01) and CD (*p*<0.01) after one year of GFD; the same profile was observed in Crohn peripheral monocytes; C) *SH2B3* expression was lower in CD versus controls (*p*=0.04) whereas it was significantly higher in Crohn and CD-GFD patients versus controls (*p*<0.01) and CD (*p*<0.01). D) *LPP* expression was lower in CD peripheral monocytes than in controls (*p*=0.04); E-F) *TNFAIP3* and *RGS1* genes expression were lower in CD peripheral monocytes versus controls (*p*<0.01) and higher in Crohn patients versus controls (*p*<0.01) and CD patients (*p*<0.01). Both genes expression levels normalized after one year of GFD;. RQ: relative quantification; Ctr: controls; CD: celiac disease; CD-GFD: celiac patients on a gluten-free diet; * *p*<0.01, **p<0.05.

*TAGAP, TNFSF14 and TNFRSF14* were expressed at similar levels in CD and CD-GFD monocytes (Figure S2). The *KIAA1109* gene was over-expressed in CD, Crohn and CD-GFD patients versus controls (*p*<0.05) (Figure 3 A). Differently*, c-REL*, and *SH2B3* expression was lower in CD monocytes than in controls, but significantly higher than in CD-GFD and Crohn monocytes (Figure 3 B and C). *LPP* expression was down-regulated in CD patients, whereas it was similar to controls in CD-GFD and Crohn patients (Figure 3 D). *TNFAIP3* mRNA expression was moderately lower in CD patients than in controls and CD-GFD patients, whereas it was over-expressed in inflamed positive controls (Figure 3 E).

The trend of *RGS1* expression was similar to that of *c-REL*, but did not differ between CD-GFD and control monocytes (Figure 3 F).

### Discriminant analysis in duodenal mucosa

To identify genes whose expression best characterizes celiac tissue versus controls, a linear discriminant equation was fitted to the standardized values of expression (RQ). By a stepwise multivariate approach 5 genes (stepwise: *TNFAIP3, IL-21, c-REL, RGS1 and LPP*) were selected for discriminating capacity (Table 1). As previously described in the methods section, by multiplying the canonical unstandardized coefficients produced by the analysis to the actual values of the RQ of the candidate genes a D-score was obtained for each individual as follows:

D-Score=(TNFAIP3*0.404)+(IL21*0.300)+(cREL*0.261)+(RGS1*0.235)+(LPP*0.222)+constant

**Table 1:** Results of the discriminant analysis in 22 controls versus 20 CD in the duodenal tissue.

**Step**	**Candidate genes**	**Wilks’ Lambda**	**Exact F**	
			**Statistic**	**p value**
1	*TNFAIP3*	0.404	59.002	<0.001
2	*IL-21*	0.300	45.521	<0.001
3	*c-REL*	0.261	35.809	<0.001
4	*RGS1*	0.235	30.143	<0.001
5	*LPP*	0.222	25.272	<0.001

The expression of five genes significantly contribute to lowering Wilks’ lambda in a stepwise process. According to this analysis, 5 genes (stepwise: TNFAIP3, IL-21, c-REL, RGS1 and LPP) were selected for discriminating capacity, with a p value always less than 0.001.

This multivariate equation discriminated efficiently CD patients from controls: 92.9% of individuals were correctly classified (95% of controls and 90.9% of CD patients). One control/20 (5%) was misclassified as a celiac and two celiacs/22 (9.1%) were misplaced as controls.

### Discriminant analysis in peripheral blood monocytes

Encouraged by the results obtained with duodenal mucosa, we developed a similar linear discriminant analysis for the gene expression observed in PBMs. We randomly divided our celiac and control cases into two balanced groups: a training set to develop the equation and a validation set to verify its efficiency. By a stepwise multivariate approach the expression of 4 candidate genes were selected with a pattern quite similar to that observes in the duodenal tissue. *LPP, c-REL, KIAA1109 and TNFAIP3* genes help to discriminate cases from controls, reaching a very low Wilks’ lambda (0.048) (Table 2). The low Wilk’s lambda obtained by this set of genes, close to 0 = complete discrimination, supports the confidence into the classification capacity of the equation in the clinical setting.

**Table 2:** Results of discriminant analysis in 18 controls and 17 CD peripheral blood monocytes.

**Step**	**Candidate genes**	**Wilks’ Lambda**	**Exact F**	
			**Statistic**	**p value**
1	*c-REL*	0.138	68.711	<0.001
2	*LPP*	0.090	50.848	<0.001
3	*TNFAIP3*	0.062	45.461	<0.001
4	*KIAA1109*	0.048	39.597	<0.001

Four genes significantly contributed to lowering Wilks’ Lambda in a stepwise process (LPP, c-REL, KIAA1109 and TNFAIP3).

**Figure 4:**
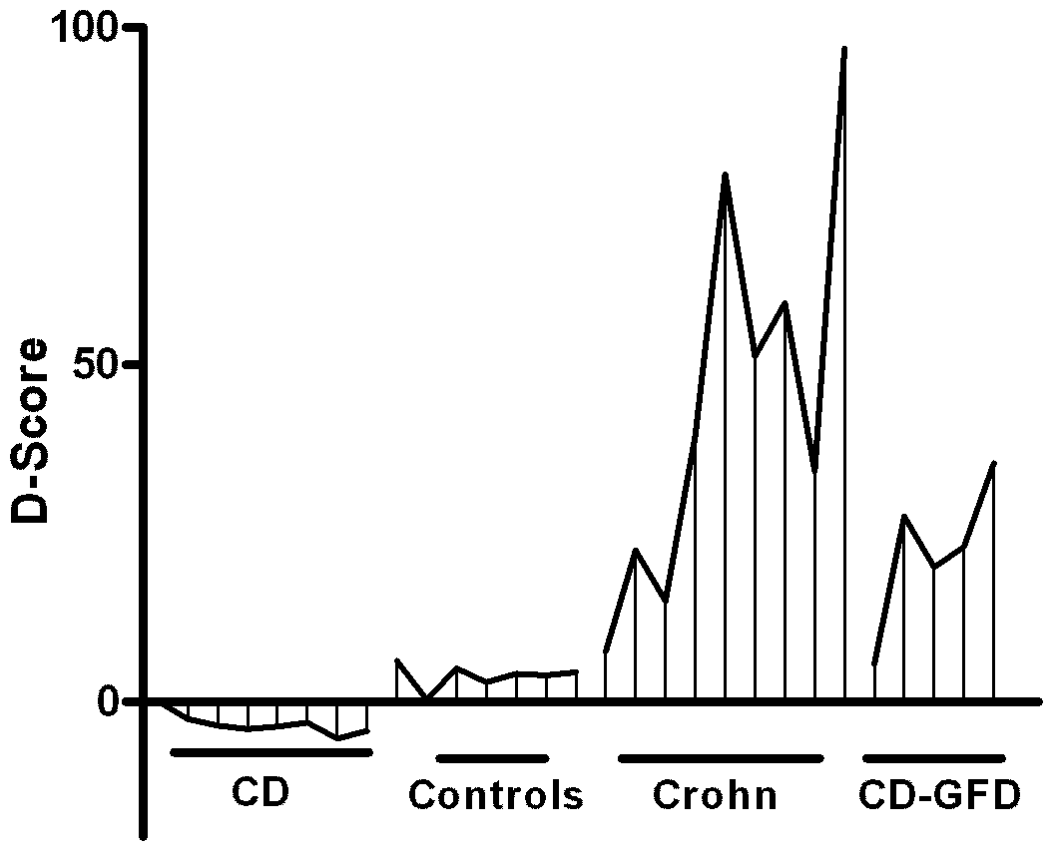
Distribution of the Discriminant Score of CD, Controls, Crohn and CD patients on gluten free diet. The D-score clearly separated the four groups of subjects evaluated. Only CD patients had a negative D-score. The D-score of CD patients on gluten free diet was intermediate between the scores of controls and Crohn patients.

Indeed 91% of controls and all CD patients were correctly classified (Table 3). To verify the efficiency of the discriminant model obtained in the training set we applied the equation to the gene expression of a new cohort of patients (validation set) made up by 7 controls, 8 CD, 5 patients on GFD and 9 disease controls (Crohn patients). We obtained four clustered D-scores, one for each group (Controls, CD, Crohn and CD on GFD) (Figure 4) with no overlap with the active celiacs. Figure 4 shows the distribution of the D-Scores of CD, CD patients on GFD, controls and Cohn’s disease patients. The D-Score of active celiac patients was negative in all cases, while it was positive for all the other groups on differentiated clusters.

**Table 3:** Classification by discriminant analysis in monocytes.

		**Predicted Group Membership**		
		**Control**	**Celiac**	**Total**
**Real Group Membership**	**Control**	10 (91%)	1 (9%)	11
	**Celiac**	0 (0%)	9 (100%)	9
	**Total**	10	10	20

By computing the discriminant score and the relative membership probability, 95.5% of patients (91% controls and 100% celiac patients) were correctly classified.

This score produces a group membership probability for each individual, allowing us to correctly classify all controls and CD patients; none of the controls, neither CD on GFD nor Crohn patients were misclassified as CD patients (Table S1).

## Discussion

As indicated in the new ESPGHAN diagnostic algorithm for CD [13], small bowel biopsy may now be avoided in a sizeable proportion of patients who have clinical symptoms: anti-tTG antibodies levels 10 times the normal values, predisposing HLA genotype. Unfortunately, not all patients have such a high production of anti-tTG antibodies, and many are asymptomatic. Moreover, this new protocol is rarely applicable to at-risk relatives [13]. Therefore, the aim of this study was to determine whether the gene expression profile of CD-associated genes in PBMs could help to differentiate patients affected by CD from controls as a step towards the molecular diagnosis of the disease. We studied the genes that have most often been associated with CD and those that show interesting functional profiles [3,4,14]. As expected, the analysis of the expression of each single gene did not unequivocally differentiate between celiac and non-celiacs, but a multivariate combination of genes that are implicated in the pathogenesis of CD was selected in the target tissue.

We previously showed that the NF-kB complex is specifically and precociously implicated in the gluten-induced inflammatory response in celiac mucosa: in fact, the NF-kB complex is fully activated just 6 hours after gluten exposure [15]. The *cREL* and TNFAIP3 genes are both involved in the regulation of this nuclear activating complex. The former is one subunit of the complex, while the latter is a negative regulator of its activation. *TNFAIP3* (also known as *A20*) expression is up-regulated by NF-kB activation, as confirmed in our study, and it acts in a negative feedback loop to control NF-kB-dependent gene expression. Furthermore, the *TNFAIP3* gene is a susceptibility locus for several human inflammatory and autoimmune diseases, including inflammatory bowel disease, rheumatoid arthritis, psoriasis, lupus and type 1 diabetes. *TNFAIP3* is also frequently inactivated in subsets of B-lineage lymphomas that are characterized by NF-kB hyper activation and was therefore suggested to be a novel tumour suppressor [16].

*IL-21* plays a crucial role in the activation of the gluten-induced mucosal activation through the NK system. It is most abundantly produced by CD4+ T cells and natural killer T (NKT) cells and is important for the development of the pro-inflammatory Th17 lineage. It has a protean function principally oriented to the activation of intraepithelial T-cell, which play a pivotal role in the development of the mucosal damage [17].

The *RGS1* gene controls the homing of intraepithelial lymphocytes (IELs), which are essential for the production of the gluten induced epithelial damage, and is less active in celiacs than in controls. It is the activation of IELs that drives the destruction of the intestinal epithelium. The main role of IELs is to promote immune protection by preventing the entry and spread of pathogens while avoiding unwanted and excessive inflammatory reactions capable of damaging the intestinal epithelium. To that end, IELs exert a cytolytic function to eliminate infected and damaged cells, and regulatory functions that contribute to epithelium healing and repair. Deregulated activation of IELs is a hallmark of CD and is critically involved in epithelial cell destruction and the subsequent development of villous atrophy. In addition, lymphocytic infiltration of the small intestinal epithelium in the absence of villous atrophy has been observed in patients with dermatitis herpetiformis [18], an autoimmune skin manifestation of CD; a finding that supports the concept that intraepithelial lymphocytosis is a marker of CD even in the absence of intestinal damage [19]. It was recently demonstrated that elevated *RGS1* levels profoundly reduce T cell migration to lymphoid-homing chemokines, whereas *RGS1* depletion selectively enhances such hemotaxis in gut T cells [20]. Its capacity to limit the egress of inflammatory and/or autoimmune cells could clearly promote immunopathology.

Finally, the *LPP* gene appears to have a relevant activity in modulating cell adhesion since it is an integral component of cell migration. It is not surprising that up- or down-regulation of *LPP* expression results in an increase or decrease, respectively, in cell migration [21]. Recent data showed that the over-expression of *LPP* increased epidermal growth factor-stimulated migration of vascular SMCs induced by TGF-β1, suggesting the participation of *LPP* in cell motility [22,23].

Since the expression of these genes is not independent, it is not possible to give a priority of function to any of them: indeed genes that are not selected by the stringent criteria of the multivariate analysis may well have their own relevant function, but their contribution is no longer significant when other more “discriminating” genes are included in the equation. Nevertheless, it is very interesting to note that this model is built by genes that are more likely to exert a relevant function in the abnormal gluten-induced response in individuals with a specific genomic profile.

When we moved from the target tissue to PBMs, to our surprise, 3 of the 4 genes selected for their discriminating capacity are the same as those included in the multivariate model of the small intestinal mucosa tissue. Again the NF-kB complex appears implicated in development of CD, as well as the fascinating *LPP* gene. The *KIAA1109* gene, located in the region encompassing *KIAA1109/Tenr/IL2/IL21* in chromosome 4q27, has often been replicated in association studies and provides a significant contribution to the discrimination between celiacs and non celiacs. This cluster region is involved in the differentiation of naïve human CD4+ T cells into Th17 cells [24]. It is important to note that Th17 cells produce a variety of cytokines, among which, *IL-17A, IL-17F, IL-21 and IL-22*. Genetic alterations in the 4q27 locus could result in non-functional *IL-21* and hence lack of *IL-17A* or vice versa. The regulation of this process may be an important factor in determining the risk for CD, as shown in such other autoimmune diseases as rheumatoid arthritis and uveitis [25,26].

In conclusion, we report an intriguing picture of the possible relationships among the expressions of candidate CD genes in the target mucosa, but also, with a minor difference, in PBMs. The analysis of the expression of each single gene is non-informative and does not reveal the specific isolated function of any of these candidates. Indeed, only the search for functional pathways may shed light on the complex gluten-induced abnormal response in genetically predisposed individuals.

We suggest that the expression of a small set of candidate genes in PBMs can be used to distinguish CD patients from healthy controls and from disease controls (patients affected by Crohn’s disease), without considering clinical data, HLA or anti-tTG antibodies. In fact, the procedure we used resulted in a distance between groups (very low Wilk’s lambda) that is unusual with ordinary diagnostic tools. We did not add anti-tTG antibodies or HLA data to the multivariate equation because it is well recognized that the former has a very high sensitivity and specificity, and the latter has a very high negative predictive value. Since we reached a correct diagnostic classification in the validation set (above 95%) by gene expression data only, we would have shown an overoptimistic estimate by adding these strong discriminators. However, this should be done in clinical practice in order to reinforce the sensitivity and the specificity of the diagnosis when duodenal biopsy is not available or desirable.

Expression may be regulated by the specific polymorphism associated to the disease but, in the case of CD, none of the identified polymorphisms contributed greatly to the pathogenesis of the gluten-induced immune response. Indeed, expression data should be examined within the framework of a reasonable pathogenic pathway. The same gene may be over- or under-expressed and produce significant downstream stimuli. Expression is one of the several regulations that control the production of functional molecules from a specific protein-coding gene. Epigenetic mechanisms have recently emerged as important partners in this domain, and our group reported a specific microRNA that is over-expressed in celiac patients versus controls [27].

Genome-wide studies have identified many encoding variants associated to CD. Several of these are implicated or are in linkage with regulatory DNA marked by deoxyribonuclease hypersensitive sites. Most of these sites are active during fetal development and associated with gestational exposure phenotype. Indeed, disease-associated variants often perturb transcription factor recognition sequences, altering the normal regulatory networks. In common human disease, and certainly in CD, regulatory DNA variations are likely to play a pivotal role, since there are no ‘missing’ or ‘failed’ genes [28].

The missing variance of heredity in CD is probably due to the thousands of expression quantitative loci, expression ‘hot spots’, where a polymorphism at a locus is responsible for changes in gene expression of many other genes, and finally by gene-by-environment interactions [29]. Gene expression is currently the best tool with which to explore the final results of genetic variance; it is quite robust and reproducible and may be tailored to specific target and non-target tissues. We cannot, therefore, predict a precise functional model of the gluten-induced immune response by studying a small, albeit important, set of genes, but we can try to obtain clues about this complexity. Interaction among genes, which is not considered in genome-wide association studies, is estimated by ordinary multivariate analysis, which is likely to provide an independent model of a possible function, or, at least, point to the genes whose expression is important in the differentiation between the affected and the unaffected.

Our discriminant function is proposed in the attempt to improve the diagnosis of CD and as a support to limit invasive techniques. Molecular analysis to discriminate a pathogenic from a healthy phenotype has become increasingly popular with the advent of innovative applications in many types of cancer and complex diseases [9]. Esophago-gastro-duodenoscopy is still the gold standard for the diagnosis of CD, but it can decrease the patient’s compliance and is indeed a major bottleneck in developing countries: a simple blood sample, which can also be easily dispatched, may help to disseminate the diagnostic coverage to the majority of patients that cannot reach a specialized reference centre [30].

In the near future, because of the new ESPGHAN protocol [13], we may have no information about the status of the traditional target tissue in many patients: gene expression on a blood sample may well add safety and sensitivity to a biopsy-free diagnostic protocol, thereby providing a good proxy of the mucosal status.

## Patients and Methods

### Ethics statement

The project was discussed in detail with parents to obtain their approval for the use of specimens collected for diagnostic proposal (small bowel biopsies and blood samples) for research purposes. Patients did not undergo specimen sampling over and above those required for routine diagnostic procedures as indicated in the ESPGHAN guidelines [31]. Written consent is included on the clinical form that the patient signs prior to collection of specimen: confidentiality between parents and doctor was an accepted proxy of a second written consent that might have been considered invasive by parents. Parents were informed of each result of the expression study that was contained in a written report included in the clinical file. The study protocol was approved by the Ethics Committee of the University of Naples “Federico II”.

### Patients

For gene expression analysis, duodenal biopsies were obtained during esophago-gastro-duodenoscopy (EGD) procedures and fresh-frozen in liquid nitrogen. Celiac disease was diagnosed according to ESPGHAN criteria, and their clinical characterization was based on the Marsh Stage classification [31]. Controls consisted of patients with a normal duodenal mucosa with no atrophy (Marsh lesion stage M0) [32].

We analyzed 48 biopsies: 20 active CD patients, 6 CD patients on a GDFD, 22 healthy controls. Controls underwent EGD because of gastritis, gastroesophageal reflux disease or suspected *Helicobacter pylori* infection. The clinical features of Crohn’s disease patients were evaluated according to the Crohn Pediatric Disease Activity Index (PDCAI) [33]. The clinical parameters of our patients are listed in Table 4 and Table 5.

**Table 4:** List of patients enrolled for analysis of duodenal biopsy expression analysis.

**Code**	**Sex**	**Age**	**Sample type**	**Clinical status**	Histology*
B6	F	15	Biopsy	CONTROL	M0
B11	F	6	Biopsy	CONTROL	M0
B12	M	14	Biopsy	CONTROL	M0
B13	F	8	Biopsy	CONTROL	M0
B14	F	7	Biopsy	CONTROL	M0
B15	M	10	Biopsy	CONTROL	M0
B17	F	12	Biopsy	CONTROL	M0
B18	F	11	Biopsy	CONTROL	M0
B22	F	9	Biopsy	CONTROL	M0
B23	M	7	Biopsy	CONTROL	M0
B24	M	8	Biopsy	CONTROL	M0
B29	F	12	Biopsy	CONTROL	M0
B30	M	14	Biopsy	CONTROL	M0
B35	F	12	Biopsy	CONTROL	M0
B36	F	10	Biopsy	CONTROL	M0
B44	M	12	Biopsy	CONTROL	M0
B46	F	13	Biopsy	CONTROL	M0
B47	M	9	Biopsy	CONTROL	M0
B48	M	10	Biopsy	CONTROL	M0
B49	M	11	Biopsy	CONTROL	M0
B50	M	8	Biopsy	CONTROL	M0
B55	M	9	Biopsy	CONTROL	M0
B1	M	7	Biopsy	CD	M3c
B2	M	5	Biopsy	CD	M3c
B3	M	11	Biopsy	CD	M3a
B7	F	9	Biopsy	CD	M3c
B8	F	12	Biopsy	CD	M3c
B9	F	11	Biopsy	CD	M3c
B10	M	11	Biopsy	CD	M3a
B16	F	8	Biopsy	CD	M3b
B19	M	13	Biopsy	CD	M3c
B21	M	11	Biopsy	CD	M3c
B25	F	9	Biopsy	CD	M3b
B26	F	13	Biopsy	CD	M3a
B27	F	9	Biopsy	CD	M3a
B34	F	10	Biopsy	CD	T3a
B37	F	6	Biopsy	CD	T3a/b
B41	F	9	Biopsy	CD	M3c
B51	F	8	Biopsy	CD	M3c
B52	F	10	Biopsy	CD	M3c
B54	F	7	Biopsy	CD	M3b
B53	F	9	Biopsy	CD	M3c
B4	F	13	Biopsy	CD-GFD	M0
B20	F	12	Biopsy	CD-GFD	M1
B28	F	14	Biopsy	CD-GFD	M1
B31	M	13	Biopsy	CD-GFD	M0
B32	M	12	Biopsy	CD-GFD	M1
B33	M	8	Biopsy	CD-GFD	M0

* For the diagnosis of CD has been applied Marsh classification, all controls have a normal duodenal mucosa with no atrophy (Marsh lesion stage M0).

**Table 5:** List of patients enrolled for analysis of peripheral blood monocyte expression analysis.

**Code**	**Sex**	**Age**	**Sample type**	**Clinical status**	Histology/disease status*†	**Cohort**
M12	F	12	Monocytes	CONTROL	M0	TRANING
M13	M	14	Monocytes	CONTROL	M0	TRANING
M14	M	7	Monocytes	CONTROL	M0	TRANING
M15	F	5	Monocytes	CONTROL	M0	TRANING
M16	F	9	Monocytes	CONTROL	M0	TRANING
M17	M	11	Monocytes	CONTROL	M0	TRANING
M18	M	9	Monocytes	CONTROL	M0	TRANING
M19	F	12	Monocytes	CONTROL	M0	TRANING
M20	M	5	Monocytes	CONTROL	M0	TRANING
M21	M	4	Monocytes	CONTROL	M0	TRANING
M22	F	8	Monocytes	CONTROL	M0	TRANING
M60	M	12	Monocytes	CONTROL	M0	VALIDATING
M61	F	5	Monocytes	CONTROL	M0	VALIDATING
M62	F	11	Monocytes	CONTROL	M0	VALIDATING
M63	M	14	Monocytes	CONTROL	M0	VALIDATING
M64	M	7	Monocytes	CONTROL	M0	VALIDATING
M65	F	11	Monocytes	CONTROL	M0	VALIDATING
M66	F	7	Monocytes	CONTROL	M0	VALIDATING
M4	M	11	Monocytes	CD	M3b	TRANING
M6	M	9	Monocytes	CD	M3c	TRANING
M7	M	7	Monocytes	CD	M3c	TRANING
M8	M	5	Monocytes	CD	M3b	TRANING
M9	M	6	Monocytes	CD	M3b/c	TRANING
M10	F	8	Monocytes	CD	M3c	TRANING
M11	M	11	Monocytes	CD	M3a	TRANING
M3	F	12	Monocytes	CD	M3c	TRANING
M5	F	7	Monocytes	CD	M3c	TRANING
M68	M	9	Monocytes	CD	M3c	VALIDATING
M69	F	10	Monocytes	CD	M3a	VALIDATING
M70	F	8	Monocytes	CD	M3c	VALIDATING
M72	M	12	Monocytes	CD	M3c	VALIDATING
M73	F	7	Monocytes	CD	M3c	VALIDATING
M74	F	5	Monocytes	CD	M3b	VALIDATING
M71	F	13	Monocytes	CD	M3c	VALIDATING
M76	M	5	Monocytes	CD	M3b/c	VALIDATING
M77	M	8	Monocytes	CD-GDF	M1	VALIDATING
M78	F	14	Monocytes	CD-GDF	M0	VALIDATING
M79	M	12	Monocytes	CD-GDF	M0	VALIDATING
M80	M	9	Monocytes	CD-GDF	M1	VALIDATING
M81	F	13	Monocytes	CD-GDF	M0	VALIDATING
C1	F	12	Monocytes	CROHN	CDAI 22,5	VALIDATING
C2	M	9	Monocytes	CROHN	CDAI 17,5	VALIDATING
C3	M	11	Monocytes	CROHN	CDAI 32,5	VALIDATING
C4	M	8	Monocytes	CROHN	CDAI 42,5	VALIDATING
C5	F	7	Monocytes	CROHN	CDAI 45	VALIDATING
C6	F	13	Monocytes	CROHN	CDAI 20	VALIDATING
C7	M	11	Monocytes	CROHN	CDAI 45	VALIDATING
C8	F	9	Monocytes	CROHN	CDAI 25	VALIDATING
C9	F	5	Monocytes	CROHN	CDAI 25,5	VALIDATING

* For the diagnosis of CD has been applied Marsh classification, all controls have a normal duodenal mucosa with no atrophy (Marsh lesion stage M0). †For Crohn’s patients is indicated disease activity index (CDAI) are accepted outcome, all patients are treated and in remission with a score of < 150.

### Monocyte isolation

We used the Dynabeads® My Pure™ Monocyte kit (Life Technologies, Foster City, CA) to isolate monocytes from other peripheral blood cell types (B- and T-lymphocytes, NK cells, erythrocytes, dendritic cells etc.). Monocytes were extracted from 10 ml of peripheral blood of 18 healthy controls, 17 CD patients, 9 Crohn’s disease patients and 5 CD patients on a GFD.

### Gene expression studies

Total RNA was extracted from duodenal biopsies and blood monocytes with the Ambion® RiboPure™ kit. The quantity of RNA was measured using the Nanodrop® spettrophotometer, and then RNA quality was analyzed by Agarose gel electrophoresis in Tris/Borate/EDTA buffer (TBE). Two µg by each biopsy, and 100 ng by monocytes of total RNA were reverse-transcribed into cDNA with the High Capacity cDNA Reverse Transcription kit, as per the manufacturer’s protocol. After retro-transcription, we carried out a linear pre-amplification step to enhance the low amount of RNA recovered from monocytes. Pre-amplification was performed with the TaqMan® PreAmp Master Mix. Experiments were performed on the 7900HT Fast Real Time PCR system using the TaqMan® Gene Expression Assay, and about 40 ng of cDNA according to the manufacturer’s protocol. The gene expression assay used for candidate genes is reported in the supplementary materials (Table S2). The relative expression was calculated with the comparative Ct method. The expression of each gene was normalized to an endogenous housekeeping gene (*GUSb*), GUSb was chosen as reference gene after it had been determined as the most stable reference gene out of 5 candidates (β-actin, B2M, GAPDH, GUSb, and HPRT1). The SDS software (ABI, version 1.4 or 2.4) was used to analyze the raw data and then an additional statistic analysis was performed on GraphPad Prism 5.01®. Relative quantification was performed using the ΔΔCt method. All gene expression experiments (original raw data available as supporting materials Table S3 and Table S4) were conducted according to MIQE guidelines (http://www.gene-quantification.de/miqe-bustin-et-al-clin-chem-2009.pdf)*.*

### Statistical analysis

The non-parametric Mann-Whitney U test for independent variables was used to assess the difference between data sets; first degree error was set at ≤0.05. ANOVA was used to estimate differences among mean expression levels, when appropriate. A discriminant analysis was performed to estimate the contribution of the expression of each gene to distinguish CD patients from healthy individuals and disease controls. The aim of this analysis is to weigh the discriminating capacity of each single gene to obtain a new composite variable, the discriminant score (D-score) which provides a group-specific score for each individual. Wilks’ lambda is an estimate of the discriminant capacity ranging from 1 (complete overlap) to 0 (maximum distance). The variable that minimizes the overall Wilks’ lambda is entered at each step. According to this analysis, only a few specific genes were selected for discriminating capacity, giving a significant contribution to the Variance ratio F, with a first degree error always less than 0.001.

By multiplying the canonical unstandardized coefficients produced by the analysis to the actual values of the RQ of the candidate genes a D-score was obtained for each individual. The discriminant score provides a probability of membership to the cases or to the controls groups for each individual. The highest membership probability for each case allows the classification into the diagnostic groups.

Statistical analyses were performed using the SPSS 17.0 (SPSS Inc., Chicago, IL, USA) and GraphPad Prism 5.0 (GraphPad software, San Diego, CA, USA) software packages.

## Supporting Information

Figure S1 Gene expression in duodenal tissue. The expression of A) LPP, B) TNFAIP3 C) RGS1 and D) TNFRSF14 genes did not differ significantly among the three groups.
(TIF)

Figure S2 Gene expression in peripheral blood monocytes. TAGAP,TNFSF14 and TNFRSF14 genes were expressed at similar levels in CD and CD-GFD monocytes.
(TIF)

Table S1 Validation of the discriminant analysis. The analysis conducted to classify controls, CD, Crohn and CD-GFD patients in 2 groups: Controls and Celiacs. The Highest Group corresponds to the first prediction choice, and the Second Highest Group to the second one. Effective control and celiac patients were all correctly predicted; Crohn and CD-GFD patients were predicted as controls.
(DOCX)

Table S2 List of TaqMan Gene Expression assays used in the expression experiments (Life Technologies, Foster City, CA).
(DOCX)

Table S3 Raw data of gene expression analysis in biopsy. *For the diagnosis of CD has been applied Marsh classification, all controls have a normal duodenal mucosa with no atrophy (Marsh lesion stage M0).
(DOCX)

Table S4 Raw data of gene expression analysis in monocytes. *For the diagnosis of CD has been applied Marsh classification, all controls have a normal duodenal mucosa with no atrophy (Marsh lesion stage M0). **†** For Crohn’s patients is indicated disease activity index (CDAI) are accepted outcome, all patients are treated and in remission with a score of < 150.
(DOCX)
