# Supplementary figures and images for: Gene Expression Profile of Peripheral Blood Monocytes: A Step towards the Molecular Diagnosis of Celiac Disease?
Source: PLoS One. 2013 Sep 17;8(9):e74747. doi: 10.1371/journal.pone.0074747 (PMC3775745; doi:10.1371/journal.pone.0074747)

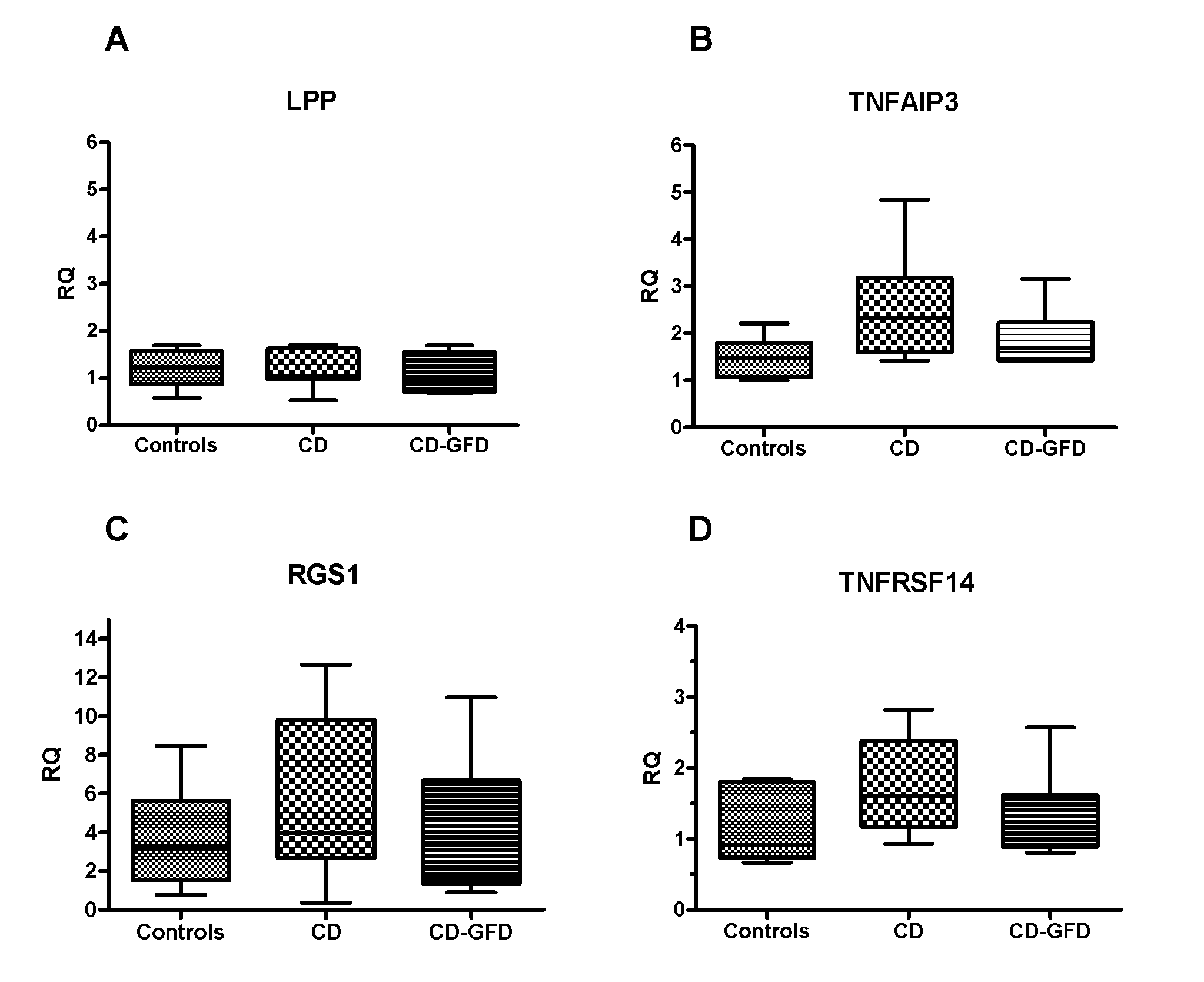

Supplement: Figure S1 — Gene expression in duodenal tissue. The expression of A) LPP, B) TNFAIP3 C) RGS1 and D) TNFRSF14 genes did not differ significantly among the three groups. (TIF) [file pone.0074747.s001.tif]

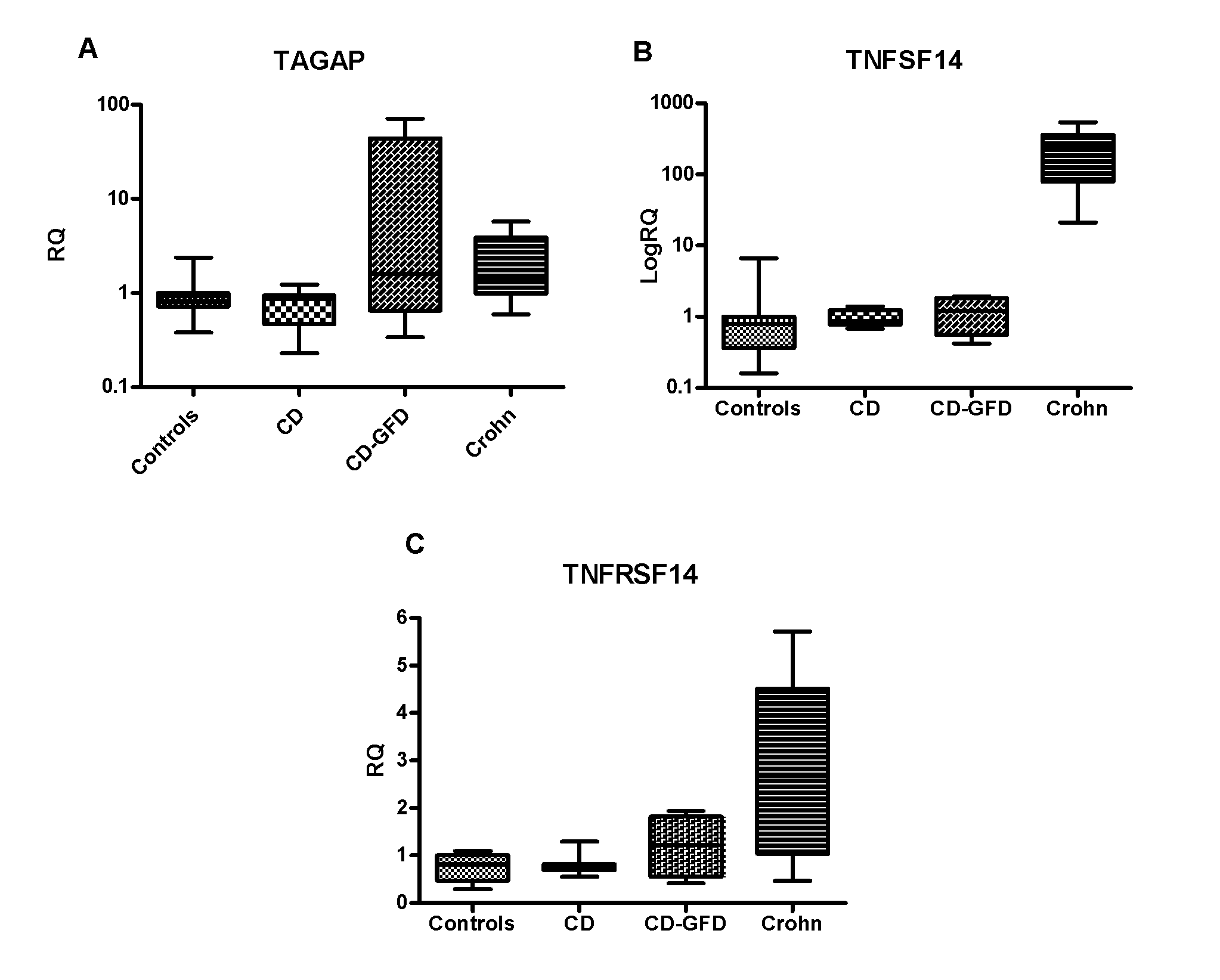

Supplement: Figure S2 — Gene expression in peripheral blood monocytes. TAGAP,TNFSF14 and TNFRSF14 genes were expressed at similar levels in CD and CD-GFD monocytes. (TIF) [file pone.0074747.s002.tif]
