# Supplementary material for: Gene Expression Profile of Peripheral Blood Monocytes: A Step towards the Molecular Diagnosis of Celiac Disease?
Source: PLoS One. 2013 Sep 17;8(9):e74747. doi: 10.1371/journal.pone.0074747 (PMC3775745; doi:10.1371/journal.pone.0074747)
Supplement: Table S1 — Validation of the discriminant analysis. The analysis conducted to classify controls, CD, Crohn and CD-GFD patients in 2 groups: Controls and Celiacs. The Highest Group corresponds to the first prediction choice, and the Second Highest Group to the second one. Effective control and celiac patients were all correctly predicted; Crohn and CD-GFD patients were predicted as controls. (DOCX) [file pone.0074747.s003.docx]

**Table S1.** Validation of the discriminant analysis. The analysis conducted to classify controls, CD, Crohn and CD-GFD patients in 2 groups: Controls and Celiacs. The Highest Group corresponds to the first prediction choice, and the Second Highest Group to the second one. Effective control and celiac patients were all correctly predicted; Crohn and CD-GFD patients were predicted as controls.

| Case Number | Actual Group | Highest Group | | | | | Discriminant Score |
| --- | --- | --- | --- | --- | --- | --- | --- |
|  |  | Predicted Group | P(D>d \| G=g) | | P(G=g \| D=d) | Squared Mahalanobis Distance to Centroid | Function 1 |
|  |  |  | p | df |  |  |  |
| 1 | Celiac | Celiac | 0.000 | 1 | 0.878 | 14.940 | 0.076 |
| 2 | Celiac | Celiac | 0.222 | 1 | 1.000 | 1.491 | -2.568 |
| 3 | Celiac | Celiac | 0.774 | 1 | 1.000 | 0.082 | -3.503 |
| 4 | Celiac | Celiac | 0.844 | 1 | 1.000 | 0.038 | -3.986 |
| 5 | Celiac | Celiac | 0.882 | 1 | 1.000 | 0.022 | -3.641 |
| 6 | Celiac | Celiac | 0.463 | 1 | 1.000 | 0.538 | -3.056 |
| 7 | Celiac | Celiac | 0.101 | 1 | 1.000 | 2.695 | -5.431 |
| 8 | Celiac | Celiac | 0.581 | 1 | 1.000 | 0.305 | -4.341 |
| 9 | Control | Control | 0.089 | 1 | 1.000 | 2.884 | 6.119 |
| 10 | Control | Control | 0.000 | 1 | 0.576 | 16.548 | 0.353 |
| 11 | Control | Control | 0.566 | 1 | 1.000 | 0.330 | 4.995 |
| 12 | Control | Control | 0.126 | 1 | 1.000 | 2.346 | 2.889 |
| 13 | Control | Control | 0.826 | 1 | 1.000 | 0.048 | 4.201 |
| 14 | Control | Control | 0.642 | 1 | 1.000 | 0.216 | 3.956 |
| 15 | Control | Control | 0.955 | 1 | 1.000 | 0.003 | 4.364 |
| 16 | Crohn | Control | 0.002 | 1 | 1.000 | 9.216 | 7.457 |
| 17 | Crohn | Control | 0.000 | 1 | 1.000 | 326.316 | 22.485 |
| 18 | Crohn | Control | 0.000 | 1 | 1.000 | 111.285 | 14.970 |
| 19 | Crohn | Control | 0.000 | 1 | 1.000 | 1232.648 | 39.530 |
| 20 | Crohn | Control | 0.000 | 1 | 1.000 | 5440.147 | 78.178 |
| 21 | Crohn | Control | 0.000 | 1 | 1.000 | 2193.662 | 51.257 |
| 22 | Crohn | Control | 0.000 | 1 | 1.000 | 2997.302 | 59.169 |
| 23 | Crohn | Control | 0.000 | 1 | 1.000 | 885.652 | 34.181 |
| 24 | Crohn | Control | 0.000 | 1 | 1.000 | 8543.655 | 96.853 |
| 25 | CD-GFD | Control | 0.232 | 1 | 1.000 | 1.431 | 5.617 |
| 26 | CD-GFD | Control | 0.000 | 1 | 1.000 | 532.145 | 27.489 |
| 27 | CD-GFD | Control | 0.000 | 1 | 1.000 | 241.667 | 19.967 |
| 28 | CD-GFD | Control | 0.000 | 1 | 1.000 | 344.648 | 22.986 |
| 29 | CD-GFD | Control | 0.000 | 1 | 1.000 | 959.294 | 35.393 |
