# Supplementary material for: Gene Expression Profile of Peripheral Blood Monocytes: A Step towards the Molecular Diagnosis of Celiac Disease?
Source: PLoS One. 2013 Sep 17;8(9):e74747. doi: 10.1371/journal.pone.0074747 (PMC3775745; doi:10.1371/journal.pone.0074747)
Supplement: Table S2 — List of TaqMan Gene Expression assays used in the expression experiments (Life Technologies, Foster City, CA). (DOCX) [file pone.0074747.s004.docx]

**Table S2.** List of TaqMan Gene Expression assays used in the expression experiments (Life Technologies, Foster City, CA).

| **Assay ID** | **Gene Symbol** | **Gene Name** | **Chromosome** | **NCBI Reference Sequence** | **Target Exons** | **Amplicon Length** | **Position** |
| --- | --- | --- | --- | --- | --- | --- | --- |
| Hs99999908_m1 | GUSB | glucuronidase, beta | 7 | NM_000181.3 | 11-12 | 81 bp | 1913 |
| Hs00611823_m1 | TAGAP | T-cell activation RhoGTPase activating protein | 6 | NM_054114.3 | 9-10 | 64 bp | 1230 |
| Hs00193878_m1 | SH2B3 | SH2B adaptor protein 3 | 12 | NM_005475.2 | 2-3 | 81 bp | 1089 |
| Hs00175260_m1 | RGS1 | regulator of G-protein signaling 1 | 1 | NM_002922.3 | 3-4 | 115 bp | 346 |
| Hs00968436_m1 | REL | v-rel reticuloendotheliosis viral oncogene homolog (avian) | 2 | NM_002908.2 | 10-11 | 86 bp | 1312 |
| Hs00234713_m1 | TNFAIP3 | tumor necrosis factor, alpha-induced protein 3 | 6 | NM_006290.2 | 2-3 | 63 bp | 363 |
| Hs00998604_m1 | TNFRSF14 | tumor necrosis factor receptor superfamily, member 14 | 1 | NM_003820.2 | 6 - 7 | 102 bp | 987 |
| Hs00222327_m1 | IL-21 | interleukin 21 | 4 | NM_001207006.2 | 3-4 | 84 bp | 406 |
| Hs00944352_m1 | LPP | LIM domain containing preferred translocation partner in lipoma | 3 | NM_001167671.1 | 8-9 | 85 bp | 1593 |
| Hs00361070_m1 | KIAA1109 | KIAA1109 | 4 | NM_015312.3 | 43-44 | 66 bp | 7360 |
| Hs00542477_m1 | TNFS14 | tumor necrosis factor (ligand) superfamily, member 14 | 19 | NM_003807.3 | 4-5 | 69 bp | 685 |
| Hs99999150_m1 | IL-2 | interleukin 2 | 4 | NM_000586.3 | 2-3 | 89 bp | 261 |
