# Supplementary material for: Gene Expression Profile of Peripheral Blood Monocytes: A Step towards the Molecular Diagnosis of Celiac Disease?
Source: PLoS One. 2013 Sep 17;8(9):e74747. doi: 10.1371/journal.pone.0074747 (PMC3775745; doi:10.1371/journal.pone.0074747)
Supplement: Table S3 — Raw data of gene expression analysis in biopsy. *For the diagnosis of CD has been applied Marsh classification, all controls have a normal duodenal mucosa with no atrophy (Marsh lesion stage M0). (DOCX) [file pone.0074747.s005.docx]

**Table S3.** Raw data of gene expression analysis in biopsy. *For the diagnosis of CD has been applied Marsh classification, all controls have a normal duodenal mucosa with no atrophy (Marsh lesion stage M0).

| **CODE** | **SEX** | **AGE** | **SAMPLE TYPE** | **CLINICAL STATUS** | **HISTOLOGY*** | **KIAA** | **IL2** | **IL21** | **LPP** | **REL** | **RGS1** | **SH2B3** | **TAGAP** | **TNFRSF14** | **TNFAIP3** | **TNFSF14** |
| --- | --- | --- | --- | --- | --- | --- | --- | --- | --- | --- | --- | --- | --- | --- | --- | --- |
| B6 | F | 15 | Biopsy | CONTROL | M0 | 1,00 | 1,00 | 1,00 | 1,56 | 0,72 | 1,99 | 2,36 | 5,40 | 0,81 | 1,09 | 1,71 |
| B11 | F | 6 | Biopsy | CONTROL | M0 | 0,91 | 1,00 | N/A | N/A | N/A | N/A | N/A | N/A | N/A | 1,55 | N/A |
| B12 | M | 14 | Biopsy | CONTROL | M0 | 1,00 | 3,15 | 1,00 | 0,97 | 1,00 | 1,23 | 3,07 | 1,89 | 0,76 | 2,21 | 1,00 |
| B13 | F | 8 | Biopsy | CONTROL | M0 | 1,32 | 5,01 | 1,25 | 1,46 | 0,86 | 1,35 | 0,98 | 1,39 | 1,12 | 1,49 | 1,62 |
| B14 | F | 7 | Biopsy | CONTROL | M0 | N/A | 1,00 | 1,12 | 1,29 | 1,00 | 7,71 | 2,23 | 2,91 | 0,67 | 1,00 | 1,00 |
| B15 | M | 10 | Biopsy | CONTROL | M0 | 1,32 | 1,00 | 0,90 | 1,00 | 0,89 | 8,47 | 1,41 | 5,61 | 1,85 | 1,49 | 2,89 |
| B17 | F | 12 | Biopsy | CONTROL | M0 | 0,77 | 2,29 | 2,95 | 0,98 | 0,72 | 4,29 | 1,32 | 2,00 | 0,95 | 1,90 | 2,70 |
| B18 | F | 11 | Biopsy | CONTROL | M0 | 1,00 | 1,00 | 1,10 | 1,64 | 0,70 | 1,72 | 3,31 | 1,75 | 1,79 | 1,47 | 1,46 |
| B22 | F | 9 | Biopsy | CONTROL | M0 | 1,00 | 2,75 | 1,25 | 1,00 | 0,66 | 1,00 | 1,00 | 2,25 | 0,67 | 1,60 | 1,18 |
| B23 | M | 7 | Biopsy | CONTROL | M0 | 1,11 | 3,00 | 1,50 | 0,72 | 0,88 | 0,78 | 0,84 | 1,00 | 1,69 | 1,37 | 1,13 |
| B24 | M | 8 | Biopsy | CONTROL | M0 | 1,19 | 2,83 | 3,00 | 0,58 | 1,10 | 2,13 | 0,85 | 1,59 | 1,00 | 1,65 | 1,00 |
| B29 | F | 12 | Biopsy | CONTROL | M0 | N/A | N/A | N/A | 1,00 | 0,89 | N/A | N/A | N/A | N/A | N/A | N/A |
| B30 | M | 14 | Biopsy | CONTROL | M0 | 1,22 | N/A | N/A | N/A | 0,75 | 3,07 | N/A | N/A | 0,77 | 1,20 | N/A |
| B35 | F | 12 | Biopsy | CONTROL | M0 | N/A | 3,17 | N/A | 1,50 | N/A | 4,74 | N/A | N/A | N/A | 1,10 | N/A |
| B36 | F | 10 | Biopsy | CONTROL | M0 | N/A | 2,77 | N/A | 0,93 | 0,53 | 7,00 | N/A | N/A | N/A | 2,10 | N/A |
| B44 | M | 12 | Biopsy | CONTROL | M0 | N/A | N/A | N/A | 1,70 | N/A | 3,05 | N/A | N/A | N/A | 2,16 | N/A |
| B46 | F | 13 | Biopsy | CONTROL | M0 | 1,34 | N/A | N/A | N/A | 0,23 | 5,50 | 2,86 | N/A | N/A | 1,50 | N/A |
| B47 | M | 9 | Biopsy | CONTROL | M0 | 1,04 | 1,00 | N/A | 0,69 | 0,47 | 4,80 | N/A | N/A | N/A | N/A | N/A |
| B48 | M | 10 | Biopsy | CONTROL | M0 | N/A | N/A | N/A | 0,85 | N/A | N/A | 1,00 | 1,59 | N/A | N/A | N/A |
| B49 | M | 11 | Biopsy | CONTROL | M0 | 0,77 | N/A | N/A | N/A | N/A | N/A | N/A | 5,31 | N/A | N/A | N/A |
| B50 | M | 8 | Biopsy | CONTROL | M0 | 1,00 | N/A | N/A | N/A | N/A | N/A | N/A | 2,71 | N/A | N/A | N/A |
| B55 | M | 9 | Biopsy | CONTROL | M0 | 1,17 | N/A | N/A | N/A | 1,20 | 3,00 | N/A | N/A | N/A | N/A | N/A |
| B1 | M | 7 | Biopsy | CD | M3c | 0,73 | 4,70 | 273,19 | 1,63 | 0,75 | 4,23 | 1,75 | 4,22 | 1,41 | 1,43 | 5,17 |
| B2 | M | 5 | Biopsy | CD | M3c | 1,68 | 4,10 | 54,87 | 1,71 | 1,60 | 3,74 | 2,84 | 5,46 | 1,94 | 2,01 | 2,34 |
| B3 | M | 11 | Biopsy | CD | M3a | 0,94 | 3,03 | 45,33 | 1,05 | 0,64 | 3,30 | 3,26 | 4,33 | 2,82 | 4,84 | 2,08 |
| B7 | F | 9 | Biopsy | CD | M3c | 0,27 | 1,25 | 17,90 | 1,70 | 0,59 | 9,80 | 1,45 | 7,41 | 2,70 | 2,63 | 2,00 |
| B8 | F | 12 | Biopsy | CD | M3c | 0,34 | 7,00 | 144,11 | 0,53 | 0,23 | 0,38 | 0,62 | 3,25 | 1,59 | 1,70 | 5,90 |
| B9 | F | 11 | Biopsy | CD | M3c | 2,65 | 2,43 | 17,25 | 1,03 | 0,74 | 0,77 | 3,98 | 3,25 | 1,60 | 1,65 | 1,81 |
| B10 | M | 11 | Biopsy | CD | M3a | 0,79 | 0,78 | 12,63 | 1,19 | 0,46 | 4,91 | 5,02 | 3,15 | N/A | N/A | N/A |
| B16 | F | 8 | Biopsy | CD | M3b | 0,96 | 7,64 | 252,90 | 1,14 | 0,46 | 9,84 | 2,31 | 2,59 | 0,93 | 2,84 | 3,25 |
| B19 | M | 13 | Biopsy | CD | M3c | 1,55 | 4,18 | 38,38 | 0,70 | 0,35 | 3,31 | 3,71 | 6,00 | 1,23 | 2,95 | 3,50 |
| B21 | M | 11 | Biopsy | CD | M3c | 1,20 | 1,32 | 78,00 | 0,97 | 0,33 | 12,64 | 2,63 | 3,33 | 1,10 | 3,80 | 2,36 |
| B25 | F | 9 | Biopsy | CD | M3b | 2,71 | 2,36 | N/A | 1,01 | 1,10 | N/A | 3,76 | N/A | 2,30 | N/A | N/A |
| B26 | F | 13 | Biopsy | CD | M3a | 0,66 | 1,91 | N/A | N/A | N/A | 5,30 | N/A | N/A | N/A | N/A | N/A |
| B27 | F | 9 | Biopsy | CD | M3a | 3,05 | 4,28 | 93,00 | N/A | 0,39 | N/A | N/A | N/A | N/A | N/A | N/A |
| B34 | F | 10 | Biopsy | CD | T3a | 1,08 | 0,31 | N/A | 1,15 | N/A | N/A | N/A | 4,30 | N/A | N/A | N/A |
| B37 | F | 6 | Biopsy | CD | T3a/b | 0,16 | 3,23 | 124,10 | 1,55 | 0,65 | N/A | 2,85 | 5,10 | N/A | 2,51 | 1,89 |
| B41 | F | 9 | Biopsy | CD | M3c | 0,34 | N/A | 66,80 | N/A | N/A | 5,10 | N/A | N/A | N/A | N/A | N/A |
| B51 | F | 8 | Biopsy | CD | M3c | 0,61 | 3,21 | N/A | N/A | N/A | N/A | 2,23 | N/A | N/A | N/A | N/A |
| B52 | F | 10 | Biopsy | CD | M3c | 0,77 | N/A | N/A | 0,65 | N/A | N/A | 3,45 | 3,50 | N/A | N/A | N/A |
| B54 | F | 7 | Biopsy | CD | M3b | 1,10 | N/A | N/A | N/A | 0,86 | N/A | N/A | N/A | N/A | N/A | N/A |
| B53 | F | 9 | Biopsy | CD | M3c | 0,98 | N/A | N/A | N/A | 1,20 | N/A | N/A | N/A | N/A | N/A | N/A |
| B4 | F | 13 | Biopsy | CD-GFD | M0 | 5,43 | 1,08 | 0,46 | 0,68 | 0,95 | 10,97 | 0,45 | 1,20 | 2,57 | 1,92 | 1,75 |
| B20 | F | 12 | Biopsy | CD-GFD | M1 | 0,79 | 1,14 | 1,86 | 0,91 | 0,81 | 1,68 | 0,35 | 1,23 | 1,30 | 1,42 | 4,27 |
| B28 | F | 14 | Biopsy | CD-GFD | M1 | 1,95 | 2,33 | 6,52 | 0,71 | 1,15 | 0,90 | 0,57 | 3,40 | 1,21 | 1,76 | 1,55 |
| B31 | M | 13 | Biopsy | CD-GFD | M0 | 1,40 | 3,40 | 0,36 | 0,93 | 0,87 | 1,46 | 0,17 | 3,70 | 1,12 | 3,16 | 2,25 |
| B32 | M | 12 | Biopsy | CD-GFD | M1 | 3,77 | 0,81 | 2,00 | 1,55 | 0,91 | 5,26 | 1,14 | 9,56 | 0,92 | 1,62 | 2,50 |
| B33 | M | 8 | Biopsy | CD-GFD | M0 | 1,75 | 2,66 | 6,00 | 1,69 | 0,79 | 1,55 | 0,72 | 4,29 | 0,81 | 1,41 | 3,70 |
