# Supplementary material for: Gene Expression Profile of Peripheral Blood Monocytes: A Step towards the Molecular Diagnosis of Celiac Disease?
Source: PLoS One. 2013 Sep 17;8(9):e74747. doi: 10.1371/journal.pone.0074747 (PMC3775745; doi:10.1371/journal.pone.0074747)
Supplement: Table S4 — Raw data of gene expression analysis in monocytes. *For the diagnosis of CD has been applied Marsh classification, all controls have a normal duodenal mucosa with no atrophy (Marsh lesion stage M0). † For Crohn’s patients is indicated disease activity index (CDAI) are accepted outcome, all patients are treated and in remission with a score of < 150. (DOCX) [file pone.0074747.s006.docx]

**Table S4.** Raw data of gene expression analysis in monocytes. *For the diagnosis of CD has been applied Marsh classification, all controls have a normal duodenal mucosa with no atrophy (Marsh lesion stage M0).**†**For Crohn’s patients is indicated disease activity index (CDAI) are accepted outcome, all patients are treated and in remission with a score of < 150.

| **CODE** | **SEX** | **AGE** | **SAMPLE TYPE** | **CLINICAL STATUS** | **HISTOLOGY/DISEASE STATUS*†** | **COHORT** | **KIAA** | **LPP** | **REL** | **RGS1** | **SH2B3** | **TAGAP** | **TNFRSF14** | **TNFAIP3** | **TNFSF14** |
| --- | --- | --- | --- | --- | --- | --- | --- | --- | --- | --- | --- | --- | --- | --- | --- |
| M12 | F | 12 | Monocytes | CONTROL | M0 | TRANING | 1 | 1 | 1 | 1 | 1 | 1 | 1 | 1 | 1 |
| M13 | M | 14 | Monocytes | CONTROL | M0 | TRANING | 1,05 | 0,98 | 0,7 | 0,81 | 0,43 | 0,96 | 0,75 | 0,81 | 0,16 |
| M14 | M | 7 | Monocytes | CONTROL | M0 | TRANING | 0,81 | 0,61 | 0,88 | 2 | 0,943 | 0,38 | 0,86 | 0,44 | 0,35 |
| M15 | F | 5 | Monocytes | CONTROL | M0 | TRANING | 1,01 | 0,92 | 0,9 | 0,799 | 0,998 | 2,36 | 0,84 | 1,15 | 0,58 |
| M16 | F | 9 | Monocytes | CONTROL | M0 | TRANING | 0,95 | 0,72 | 0,78 | 0,973 | 0,599 | 0,49 | 0,29 | 1,41 | 0,64 |
| M17 | M | 11 | Monocytes | CONTROL | M0 | TRANING | 0,79 | 1,06 | 0,78 | 0,891 | 1,2 | 1 | 0,53 | 1,1 | 0,36 |
| M18 | M | 9 | Monocytes | CONTROL | M0 | TRANING | 1,16 | 1,1 | 0,82 | 1,127 | 0,96 | 1,02 | 0,42 | 0,88 | 0,36 |
| M19 | F | 12 | Monocytes | CONTROL | M0 | TRANING | 0,75 | 0,95 | 0,81 | 1,375 | 0,73 | 0,7 | 0,64 | 0,87 | 1,02 |
| M20 | M | 5 | Monocytes | CONTROL | M0 | TRANING | 1,01 | 0,89 | 0,88 | 0,852 | 0,91 | 0,73 | 1,09 | 0,95 | 0,92 |
| M21 | M | 4 | Monocytes | CONTROL | M0 | TRANING | 0,9 | 0,71 | 1,1 | 0,965 | 0,954 | 1,399 | 0,54 | 0,99 | 1,7 |
| M22 | F | 8 | Monocytes | CONTROL | M0 | TRANING | 0,82 | 1,11 | 1,83 | 6,85 | 1 | 0,971 | 0,82 | 0,89 | 1,18 |
| M60 | M | 12 | Monocytes | CONTROL | M0 | VALIDATING | 1 | 1 | 1 | 1 | 1 | 0,95 | 1 | 1 | 1 |
| M61 | F | 5 | Monocytes | CONTROL | M0 | VALIDATING | 0,54 | 1,36 | 0,64 | 0,45 | 2,3 | 0,85 | 0,42 | 0,69 | 0,96 |
| M62 | F | 11 | Monocytes | CONTROL | M0 | VALIDATING | 1,09 | 1,11 | 0,67 | 3,75 | 2,07 | 0,82 | 1,04 | 0,37 | 0,38 |
| M63 | M | 14 | Monocytes | CONTROL | M0 | VALIDATING | 0,84 | 0,57 | 1,54 | 0,66 | 1,23 | 0,98 | 0,41 | 1,41 | 2,36 |
| M64 | M | 7 | Monocytes | CONTROL | M0 | VALIDATING | 0,88 | 0,28 | 0,53 | 2,13 | 0,64 | 0,87 | 1,02 | 2,24 | 0,49 |
| M65 | F | 11 | Monocytes | CONTROL | M0 | VALIDATING | 0,35 | 0,41 | 1,48 | 0,65 | 1,29 | 1,1 | 1 | 2,78 | 6,61 |
| M66 | F | 7 | Monocytes | CONTROL | M0 | VALIDATING | 0,62 | 0,49 | 1,1 | 0,95 | 1,35 | 1,2 | 0,5 | 2,96 | 1,12 |
| M4 | M | 11 | Monocytes | CD | M3b | TRANING | 0,68 | 0,6 | 1,1 | 0,83 | 0,62 | 1 | 0,43 | 0,76 | 0,68 |
| M6 | M | 9 | Monocytes | CD | M3c | TRANING | 0,85 | 0,49 | 0,38 | 0,22 | 0,77 | 0,92 | 0,76 | 1,5 | 0,85 |
| M7 | M | 7 | Monocytes | CD | M3c | TRANING | 0,72 | 0,37 | 0,29 | 0,28 | 0,45 | 0,59 | 0,64 | 0,78 | 0,72 |
| M8 | M | 5 | Monocytes | CD | M3b | TRANING | 1,11 | 0,65 | 0,41 | 0,46 | 0,7 | 0,92 | 0,55 | 0,69 | 1,11 |
| M9 | M | 6 | Monocytes | CD | M3b/c | TRANING | 1,29 | 0,47 | 0,45 | 0,53 | 0,43 | 0,86 | 0,79 | 1,05 | 1,29 |
| M10 | F | 8 | Monocytes | CD | M3c | TRANING | 0,81 | 0,36 | 0,33 | 0,5 | 0,51 | 0,5 | 0,87 | 0,27 | 1,36 |
| M11 | M | 11 | Monocytes | CD | M3a | TRANING | 0,75 | 0,59 | 0,39 | 0,04 | 0,41 | 0,89 | 0,72 | 0,69 | 0,81 |
| M3 | F | 12 | Monocytes | CD | M3c | TRANING | 1,4 | 0,39 | 0,35 | 0,02 | 0,22 | 0,331 | 0,75 | 0,45 | 0,75 |
| M5 | F | 7 | Monocytes | CD | M3c | TRANING | 0,78 | 0,52 | 0,55 | 0,02 | 0,75 | 0,35 | 0,71 | 0,51 | 1,4 |
| M68 | M | 9 | Monocytes | CD | M3c | VALIDATING | 0,65 | 0,57 | 0,9 | 0,47 | 1,94 | 0,23 | 0,76 | 1,81 | 0,78 |
| M69 | F | 10 | Monocytes | CD | M3a | VALIDATING | 1,11 | 0,52 | 0,47 | 2,81 | 1,06 | 0,87 | 1300 | 0,51 | 0,87 |
| M70 | F | 8 | Monocytes | CD | M3c | VALIDATING | 0,83 | 0,79 | 0,37 | 0,55 | 1,05 | 0,82 | 0,59 | 2,54 | 0,82 |
| M72 | M | 12 | Monocytes | CD | M3c | VALIDATING | 1,64 | 0,46 | 0,37 | 0,05 | 0,69 | 1 | 0,7 | 0,42 | 1 |
| M73 | F | 7 | Monocytes | CD | M3c | VALIDATING | 1,55 | 0,62 | 0,35 | 0,59 | 1,33 | 1,22 | 0,62 | 0,67 | 1,22 |
| M74 | F | 5 | Monocytes | CD | M3b | VALIDATING | 1,63 | 0,79 | 0,57 | 1,86 | 0,32 | 0,97 | 0,52 | 0,24 | 0,9 |
| M71 | F | 13 | Monocytes | CD | M3c | VALIDATING | 1,5 | 0,98 | 0,38 | 0,49 | 0,33 | 1 | 0,8 | 0,52 | 0,74 |
| M76 | M | 5 | Monocytes | CD | M3b/c | VALIDATING | 0,86 | 0,6 | 0,27 | 0,47 | 0,44 | 0,75 | 0,67 | 1,3 | 0,69 |
| M77 | M | 8 | Monocytes | CD-GDF | M1 | VALIDATING | 0,81 | 0,75 | 0,41 | 0,296 | 2,296 | 1,586 | 1,489 | 0,44 | 1,489 |
| M78 | F | 14 | Monocytes | CD-GDF | M0 | VALIDATING | 0,79 | 0,8 | 3,32 | 6,243 | 2,036 | 0,338 | 0,945 | 1,83 | 0,945 |
| M79 | M | 12 | Monocytes | CD-GDF | M0 | VALIDATING | 1,1 | 1 | 2,43 | 0,272 | 2,265 | 0,964 | 1,936 | 1,16 | 1,936 |
| M80 | M | 9 | Monocytes | CD-GDF | M1 | VALIDATING | 1,31 | 1,12 | 2,69 | 1,521 | 3,178 | 71,138 | 0,42 | 0,57 | 0,42 |
| M81 | F | 13 | Monocytes | CD-GDF | M0 | VALIDATING | 1,54 | 0,654 | 4,28 | 1,242 | 2,8 | 17 | 1,12 | 1,04 | 0,98 |
| C1 | F | 12 | Monocytes | CROHN | CDAI 22,5 | VALIDATING | 0,17 | 0,45 | 1,6 | 1,68 | 0,6 | 1,39 | 0,91 | 1,02 | 21 |
| C2 | M | 9 | Monocytes | CROHN | CDAI 17,5 | VALIDATING | 0,42 | 0,64 | 2,7 | 3,14 | 0,44 | 0,59 | 3,45 | 0,98 | 108 |
| C3 | M | 11 | Monocytes | CROHN | CDAI 32,5 | VALIDATING | 0,51 | 0,26 | 0,83 | 2,5 | 1,95 | 0,75 | 0,47 | 4,48 | 201 |
| C4 | M | 8 | Monocytes | CROHN | CDAI 42,5 | VALIDATING | 1,22 | 1,9 | 4,1 | 0,82 | 0,66 | 3,89 | 1,18 | 3 | 279 |
| C5 | F | 7 | Monocytes | CROHN | CDAI 45 | VALIDATING | 2,24 | 0,85 | 7,2 | 2,46 | 1,82 | 3,62 | 4,42 | 1,25 | 350 |
| C6 | F | 13 | Monocytes | CROHN | CDAI 20 | VALIDATING | 1,24 | 0,55 | 5,4 | 11,095 | 4,16 | 5,754 | 5,71 | 3,22 | 375 |
| C7 | M | 11 | Monocytes | CROHN | CDAI 45 | VALIDATING | 1,52 | 0,98 | 6 | 6,819 | 2,21 | 0,98 | 4,6 | 7,03 | 50 |
| C8 | F | 9 | Monocytes | CROHN | CDAI 25 | VALIDATING | 2,33 | 1,15 | 3,4 | 3,09 | 2,71 | 1,495 | 1,65 | 4,06 | 539 |
| C9 | F | 5 | Monocytes | CROHN | CDAI 25,5 | VALIDATING | 2,9 | 1,35 | 8,7 | 6,25 | 2,58 | 1,2 | 2,68 | 2,48 | 250 |
